# The ABD on the nascent polypeptide and PH domain are required for the precise Anillin localization in *Drosophila* syncytial blastoderm

**DOI:** 10.1038/s41598-018-31106-0

**Published:** 2018-08-27

**Authors:** Tomoki Hirashima, Ryo Tanaka, Masamitsu Yamaguchi, Hideki Yoshida

**Affiliations:** 1 Department of Applied Biology, Kyoto Institute of Technology, Matsugasaki, Sakyo-ku, Kyoto 606-8585 Japan; 2 The Center for Advanced Insect Research, Kyoto Institute of Technology, Matsugasaki, Sakyo-ku, Kyoto 606-8585 Japan

**Keywords:** Cell division, Embryogenesis

## Abstract

Targeting proteins to regions where they are required is essential for proper development of organisms. For achievement of this, subcellular mRNA localization is one of the critical mechanisms. Subcellular mRNA localization is an evolutionarily conserved phenomenon from *E*. *coli* to human and contributes to limiting the regions at which its products function and efficiently supplies substrates for protein translation. During early *Drosophila* embryogenesis, while 71% of the 3370 mRNAs analyzed have shown prominent subcellular localization, the underlying molecular mechanisms have not been elucidated. Here, we reveal that *anillin* mRNA, one of the localized mRNAs in early *Drosophila* embryo, localizes to the tip of the pseudo-cleavage furrow in the *Drosophila* syncytial blastoderm using *in situ* hybridization combined with immunohistochemistry. Localization analyses with transgenic fly lines carrying a series of deletion mRNAs indicate that this localization is dependent on its own nascent polypeptides including the actin binding domain (ABD). In addition to the mRNA localization, it is revealed that the pleckstrin homology (PH) domain of Anillin protein is also required for its proper localization. Thus, we indicate that the precise localization of Anillin protein is tightly regulated by the ABD on the nascent polypeptide and PH domain in the *Drosophila* syncytial blastoderm.

## Introduction

**Figure 1:**
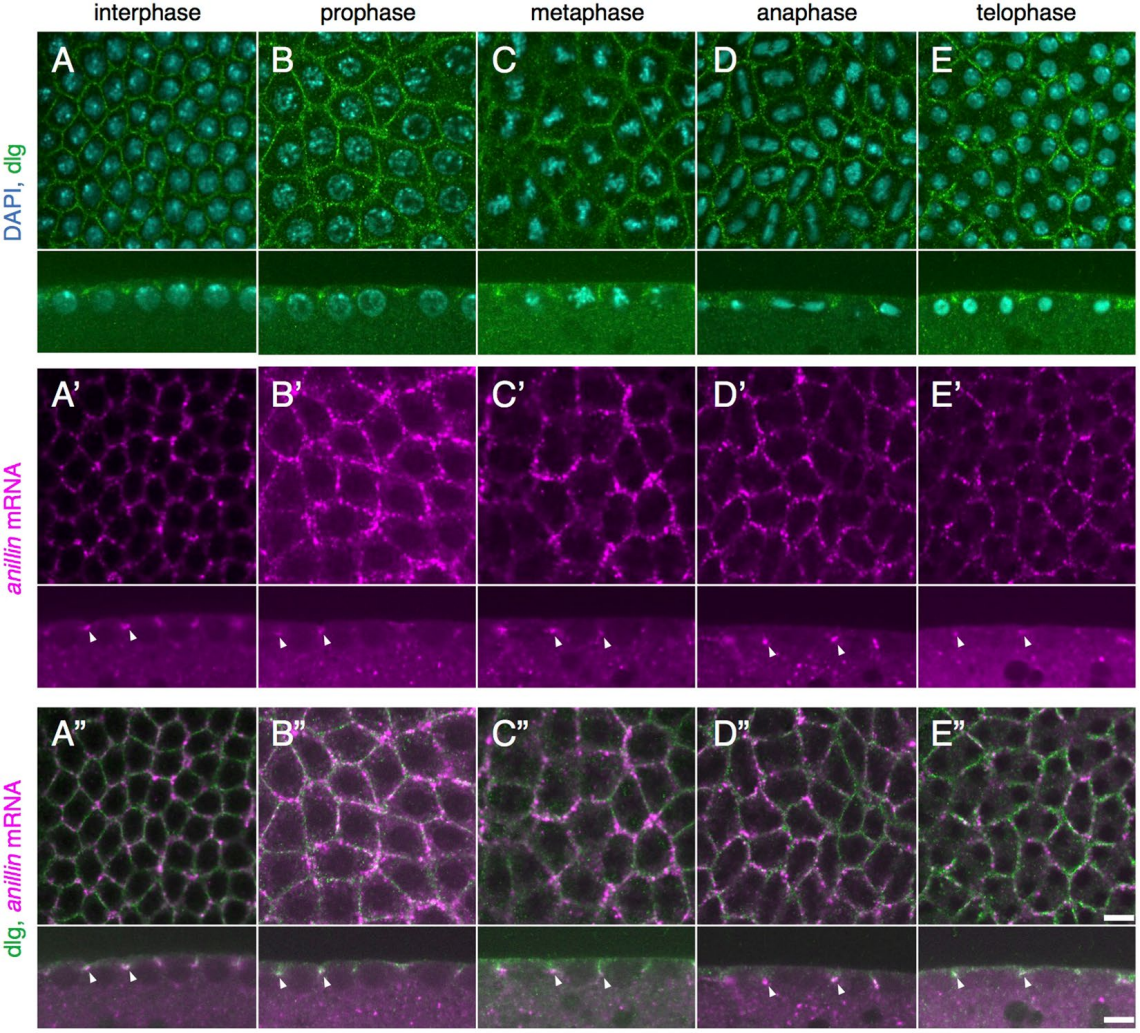
Endogenous *anillin* mRNA localizes to the tip of the PCF through the cell cycle in the syncytial blastoderm. (**A**–**E**) The plasma membrane of the syncytial blastoderm at interphase, prophase, metaphase, anaphase, and telophase was marked with anti-dlg IgG and DNA was stained with DAPI. (A’–E’) FISH images with *anillin* mRNA probes. *anillin* mRNA was detected in the syncytial blastoderm by *in situ* hybridization with the DIG-labeled RNA probe against *anillin* mRNA (arrowheads in the lower panels of A’–E’). (A”–E”) Merged images of *anillin* mRNA signals and dlg signals. *anillin* mRNA localized to the tip of the PCF, an invagination of the plasma membrane, throughout the cell cycle (arrowheads in the lower panels of A”–E”). The localization of *anillin* mRNA at the tip of the PCF in at least three different embryos was observed. There was no significant difference among these three embryos. Each upper and lower panel shows horizontal and vertical sections, respectively. Each panel is shown at the same magnification. The scale bar indicates 50 µm.

In early *Drosophila* embryogenesis, the first thirteen nuclear divisions occur without accompanying cytokinesis^1^. In the embryo between nuclear division cycle 10 and 14, which is called the syncytial blastoderm, thousands of nuclei at the embryo surface rapidly and synchronously divide four times. In order to prevent the collision of neighboring mitotic spindles, a transient membrane invagination called the pseudo-cleavage furrow (PCF) or metaphase furrow occurs between nuclei^2,3^. This furrow progressively ingresses from prophase to metaphase, and then starts to retract at anaphase (Fig. 1A–E)^4^. Most of the components in the PCF are shared with those of the cleavage furrow during cytokinesis^5–10^. The Anillin protein is one of the main components in the cleavage furrow and functions as a scaffold protein to maintain the contractile ring during cytokinesis and also localizes to the PCF in the syncytial blastoderm with strong accumulation at the tip of the PCF^7^. However, the mechanisms by which the Anillin protein is localized to the location at which it functions remain unclear.

Subcellular mRNA localization provides the appropriate amount of a protein at the region at which it is required. Since subcellularly localized mRNA was initially visualized in Ascidian embryos in 1983^11^, a large number of localized mRNAs have been reported in various species and types of cells (Holt and Bullock, 2009). The *Drosophila* embryo is an attractive model in analyses of subcellular mRNA localization, and has been utilized for more than three decades. A number of developmentally important genes have been identified and characterized in the *Drosophila* embryo, and, thus, the subcellular localization of their mRNAs may be directly linked to their functions, as is the case for *bicoid* and *nanos* mRNAs (Ephrussi *et al*., 1991; Gavis and Lehmann, 1992). Furthermore, a large-scale analysis of mRNA localization in the *Drosophila* early embryo by fluorescence *in situ* hybridization has been conducted and several types of prominent mRNA localization patterns have been reported, such as accumulation in the posterior yolk or pole plasm, apical localization above nuclei of the embryo surface, and nuclear division-related localization^12^.

In the present study, we focus on *anillin* mRNA, which shows a hexagonal pattern that is one of the distinguishing patterns in the syncytial blastoderm, and this category includes several mRNAs such as *canoe*, *Diaphanous*, *Polychaetoid*, and *CG43427* ^12^. In order to investigate the molecular mechanisms underlying the subcellular localization of *anillin* mRNA in the syncytial blastoderm, we established transgenic fly lines expressing *anillin* mRNAs carrying a set of deletions and analyzed their localization using *in situ* hybridization analysis. In the present study, we reveal that *anillin* mRNA localized to the tip of the PCF in the syncytial blastoderm in its own nascent polypeptide-dependent manner. In addition to the mRNA localization, the pleckstrin homology domain of Anillin protein is required for its proper localization at the tip of the PCF in the syncytial blastoderm.

## Results

### *anillin* mRNA localizes to the tip of the PCF throughout the cell cycle in the syncytial blastoderm

The diagnostic localization of the *anillin* mRNA like honeycomb-like pattern in the *Drosophila* syncytial blastoderm^12^ closely resembles the pattern of the PCF, which is an incomplete and temporal membrane invagination^2,3,13^. In addition, *anillin* mRNA partially colocalizes with the Anillin protein in the plasma membrane, particularly at the tip of the PCF in the syncytial blastoderm (Supplementary Fig. 1, arrowheads in lower panels)^7,12^. These findings strongly suggest that *anillin* mRNA localizes to the PCF. In order to investigate more precisely whether *anillin* mRNA localizes to the PCF, we performed *in situ* hybridization combined with immunostaining with anti-discs large (dlg) IgG, a plasma membrane and PCF marker (Lee *et al*., 2003; Cao *et al*.^10^. In the syncytial blastoderm, the cell cycle consists of interphase and a mitotic phase without gap phases and progresses quickly (Zalokar and Erk, 1976; Foe and Alberts, 1983). The results of double staining showed that *anillin* mRNA partially colocalized to the front edge of the dlg signal throughout the cell cycle in the syncytial blastoderm (Fig. 1, arrowheads in the lower panels of A”–E”).

### The coding region of a splicing variant of *anillin* mRNA, *anillin* SV-A is necessary for its localization at the tip of the PCF

**Figure 2:**
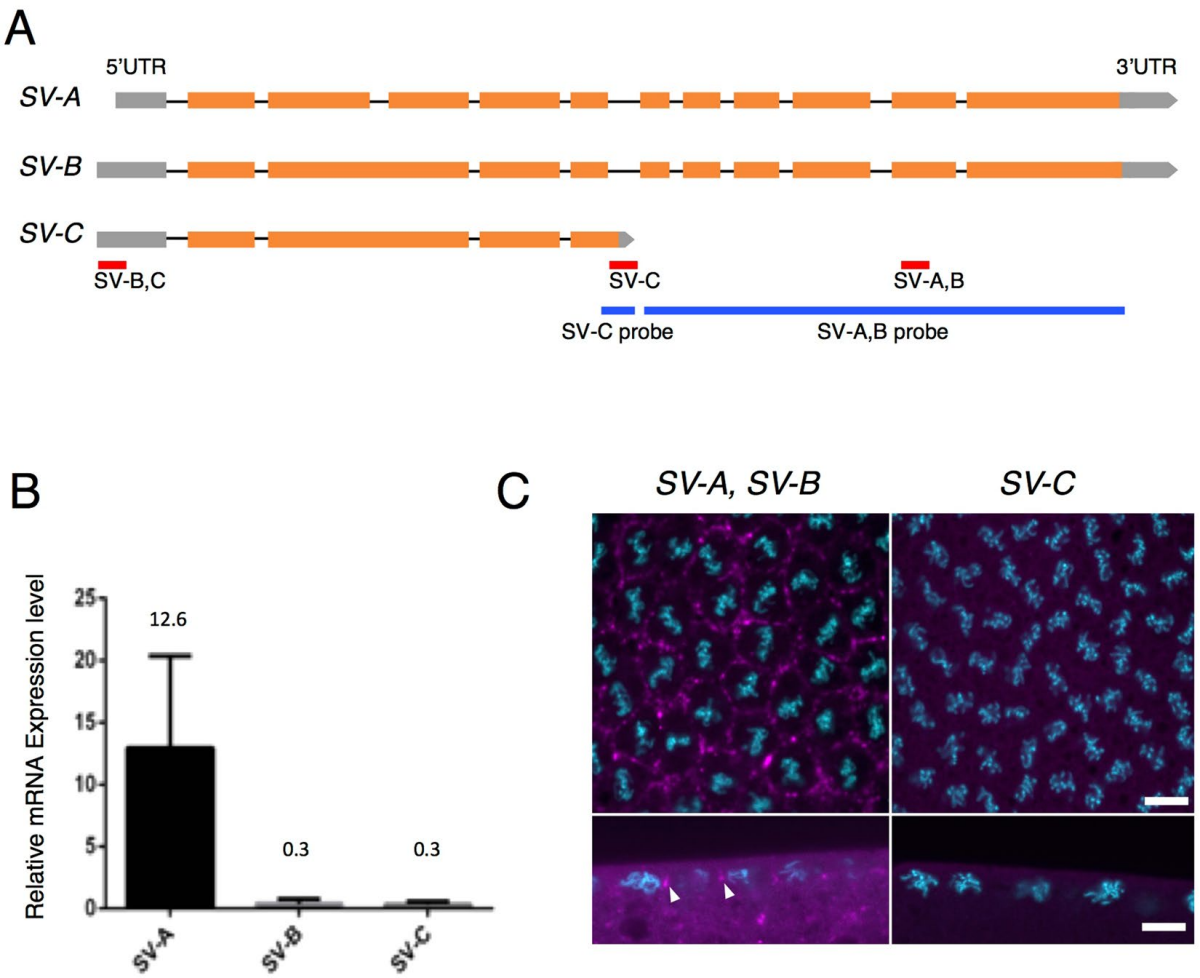
*SV-A* is dominantly expressed and localized to the tip of the PCF. (**A**) Diagram of three different predicted SVs of *anillin* mRNA. Gray box: 5′UTR, Orange box: exon, Gray pentagon: 3′UTR, Black bar: intron, Red bar: the amplified region for detecting each SV, Blue bar: the region at which each probe hybridizes for FISH. (**B**) The expression level of each *SV* was detected by RT-qPCR. Only *SV-A* was expressed at a high level in the syncytial blastoderm. Data are means ± SD (n = 3). (**C**) The localization patterns of the *SVs* that hybridized with the *SV-A*, *B* and *SV-C* probes. The signals of *SVs* are shown in magenta and DNA stained with DAPI is shown in cyan. The localization pattern of each *SV* in at least three different embryos was observed. There was no significant difference among the examined embryos. Upper and lower panels show horizontal and vertical sections, respectively. Each panel is shown at the same magnification. The scale bar indicates 50 µm.

According to the database (Flybase: http://flybase.org/reports/FBgn0261385.html), three different splice variants (*SV-A*, *B* and *C*) of *anillin* mRNA have been predicted to exist (Fig. 2A). In order to identify the PCF localization element in *anillin* mRNA, we initially investigated which SV of *anillin* mRNA was expressed and localized to the PCF in the syncytial blastoderm. We measured the expression levels of three SVs using RT-qPCR. Since it was not possible to detect *SV-A* or *B* independently (Fig. 2A), we calculated the expression level of *SV-A* or *B* based on differences between the expression levels of *SV-A*, *B* or *SV-B*, *C* and *SV-C*, respectively. RT-qPCR revealed that the expression level of *SV-A* in the syncytial blastoderm was 42-fold higher than those of *SV-B* and *C*, which were close to the limit of detection by this method (Fig. 2B). In addition to the quantitative analysis, we examined the localization of each SV by *in situ* hybridization. We were unable to detect any signals of *SV-C* (Fig. 2C). Similar to the quantitative analysis, we were unable to find a probe with the ability to detect *SV-A* and *B* separately (Fig. 2A). However, the signals observed appeared to represent *SV-A* signals because *SV-A* was dominantly expressed in the syncytial blastoderm (Fig. 2B). *SV-C* was expressed as weakly as *SV-B* and, thus, a signal for *SV-C* was not detected (Fig. 2C). These results indicate that most of the *anillin* mRNA signals at the tip of the PCF are derived from *SV-A* signals (Fig. 2C and Supplementary Fig. 2A).

**Figure 3:**
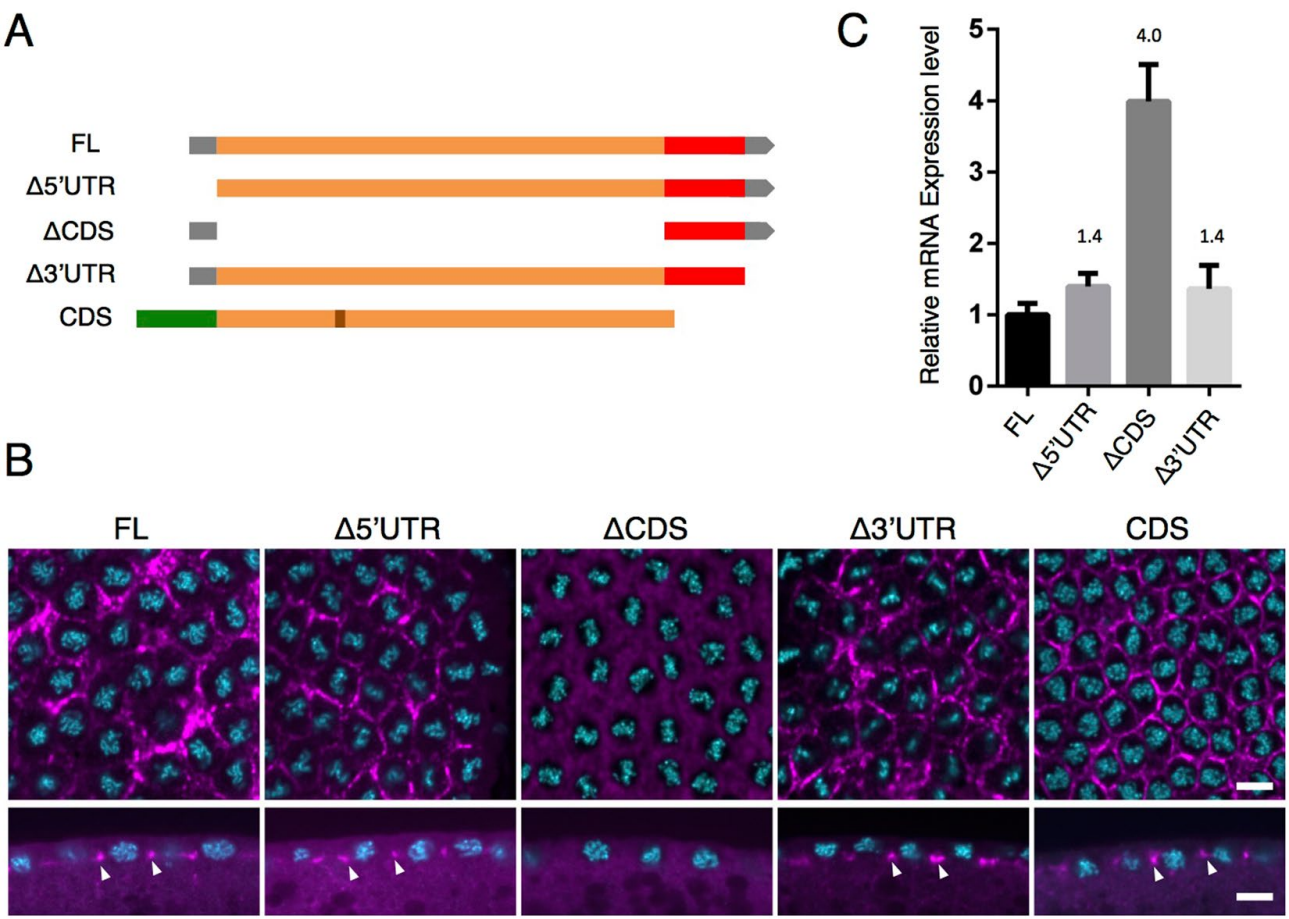
The CDS of *SV-A* is necessary and sufficient for the proper localization of *SV-A*. (**A**) Diagrams of deletion constructs of *anillin* fused with the DsRed-monomer or GFP coding sequence. FL, Δ5′UTR, ΔCDS and Δ3′UTR were derived from *SV-A* whereas CDS was derived from *SV-B* carrying additional 81 bp comparing with *SV-A*. Gray box: 5′UTR, Orange box: exon, Gray pentagon: 3′UTR, Red box: DsRed-monomer-coding sequence, Green box: GFP-coding sequence, Brawn box: additional 81 bp. (**B**) The localization pattern of each deletion derivative of mRNA detected with an anti-sense RNA probe against the DsRed-monomer-coding sequence. Each deletion mRNA is shown in magenta and DNA stained with DAPI is shown in cyan. The localization pattern of each mRNA in at least three different embryos was observed. There was no significant difference among the examined embryos. Upper and lower panels show horizontal and vertical sections. Each panel is shown at the same magnification. The scale bar indicates 50 µm. (**C**) The expression level of each deletion construct was detected by RT-qPCR. *ΔCDS* was more strongly expressed than FL and other deletion derivatives. Data are means ± SD (n = 3).

In order to identify the region in *SV-A* that is necessary for localization to the tip of the PCF, we established transgenic fly lines expressing a series of deletion derivatives of *SV-A* fused to *DsRed-monomer* (Fig. 3A). We performed *in situ* hybridization with the probe for *DsRed-monomer* to identify exogenous RNAs without detecting endogenous *anillin* mRNA. While 5′ or 3′ untranslated region (UTR)-deleted mRNAs (*Δ*5′*UTR* and *Δ*3′*UTR*) localized to the tip of the invaginating furrow, as observed with *FL* (Fig. 3B, arrowheads and Supplementary Fig. 2B), coding sequence (CDS)-deleted RNA (*ΔCDS*) did not and was evenly distributed throughout the embryo (Fig. 3B, *Δ*CDS and Supplementary Fig. 2B). RT-qPCR analyses revealed that *ΔCDS* RNA was sufficiently expressed to be detectable even if it was not specifically localized because the expression level of *ΔCDS* RNA was found to be 4.0-fold higher than that of the PCF-localized *FL* (Fig. 3C). These results suggest that CDS is crucial for the furrow tip localization of *anillin* mRNA in the syncytial blastoderm. In order to further demonstrate the importance of the CDS region of *anillin* mRNA for localization to the tip of the PCF, we investigated the localization of *GFP*-fused *anillin CDS* ^14^ using *in situ* hybridization with the probe for *GFP*. We used the transgenic strain carrying GFP-fused *SV-B* (*CDS*), which is available from a stock center. CDS is derived from *SV-B*, which includes all *SV-A* CDS and additional 81-bp sequences (Fig. 2A), and the SV-B protein has been reported to properly localize at the PCF in the syncytial blastoderm^14^. The results obtained showed that *CDS* mRNA localized to the tip of the PCF (Fig. 3B). Collectively, these results suggest that the CDS region of *anillin* mRNA is necessary and sufficient for localization to the tip of the PCF in the syncytial blastoderm (Fig. 3C).

In order to further narrow down the region required for the furrow tip localization of *anillin* CDS, we divided *SV-A* CDS into three parts (Supplementary Fig. 3A) and generated transgenic fly lines expressing each divided CDS. None of the *SV-A* carrying each of the three parts of *CDS* mRNAs localized to the PCF (Supplementary Figs 3C–E and 2C), despite all of them being sufficiently expressed for detection by RT-qPCR (Supplementary Fig. 3F). These results indicate that none of the segments for *CDS* mRNA were sufficient for localization to the furrow tip.

However, the proteins encoded by each divided CDS segment showed different localization patterns, such as on the plasma membrane, in the nucleus, or colocalization with Peanut (Supplementary Fig. 3C”–E”). These localization patterns appeared to depend on the domain feature carried in each CDS segment. There are well-known domains in each CDS segment of Anillin, such as the myosin binding domain (MBD) and actin binding domain (ABD) in CDS 1, nuclear localization signal (NLS) in CDS 2 and NLS, the pleckstrin homology (PH) domain, which binds to Peanut, and amino acids in the PH domain that are critical for PI(4,5)P_2_ binding in CDS 3^7,14–16^. The localization pattern of each CDS segment was consistent with those predicted from the function of each domain included in each CDS segment (Supplementary Fig. 3C’–E’).

### *anillin* mRNA localizes to the tip of the PCF in a manner that is dependent on its nascent polypeptides

**Figure 4:**
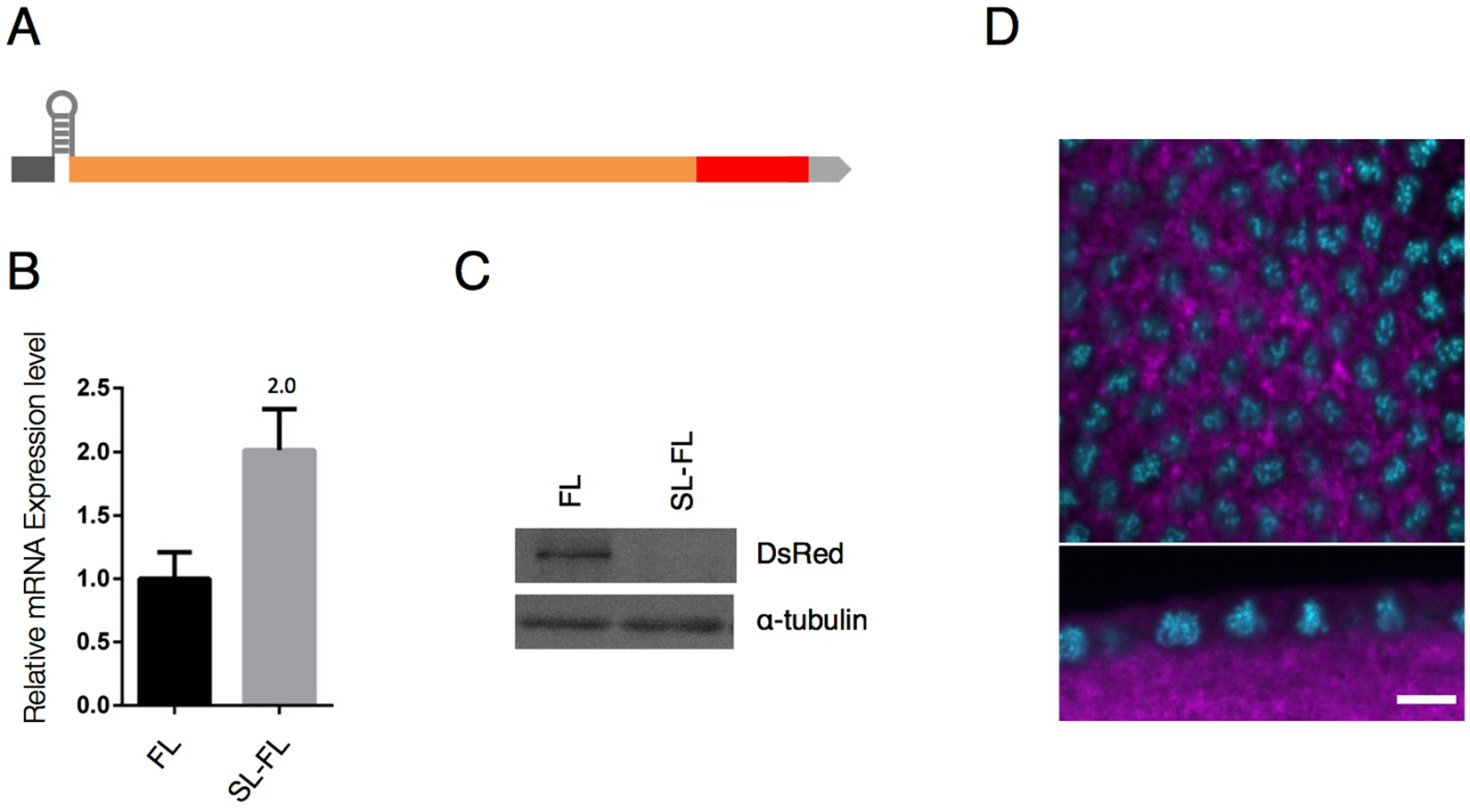
Translation of *anillin* mRNA is necessary for its localization to the tip of the PCF. (**A**) Diagram of a construct in which a stable stem-loop was inserted between *5*′*UTR* and *CDS* of *FL* (SL-FL). (**B**) *FL* mRNA and *SL-FL* mRNA levels were quantified by RT-qPCR. *SL-FL* mRNA expression levels were 2-fold higher than those of *FL* mRNA. Data are means ± SD (n = 3). (**C**) The SL-FL protein was not detected in a Western blot analysis. (**D**) *SL-FL* mRNA did not localize to the tip of the PCF. mRNA is shown in magenta and DNA stained with DAPI is shown in cyan. Upper and lower panels show horizontal and vertical sections. The scale bar indicates 50 µm.

In divided CDS segment localization analyses, we were unable to identify any obvious RNA localization element within *anillin CDS*. Previous studies reported that the mRNAs encoding transmembrane or secreted proteins localized to the endoplasmic reticulum (ER) in a manner that was mediated by their own nascent polypeptides^17^. Furthermore, as is the case with these mRNAs, some of the mRNAs encoding cytoplasmic or nuclear proteins, such as *X-box binding protein 1*, *Diaphanous 1*, and *ABP140* mRNAs, also localized subcellularly depending on their nascent polypeptides^18–20^. In order to investigate whether the localization of *anillin* mRNA depends on its own nascent polypeptides, we established transgenic fly lines harboring full-length *anillin* mRNA in which a stable stem-loop structure (∆G = −42.10 kcal/mol) was inserted between 5′UTR and CDS (*SL-FL*) to inhibit protein translation (Fig. 4A). This stem-loop structure is commonly used to prevent translation by impairing ribosome loading onto CDS^21,22^. We found that while the expression levels of *SL-FL* were 2-fold higher level than those of *FL* mRNA (Fig. 4B), no predicted protein was detected in the SL-FL fly early embryo (Fig. 4C). Under these conditions, *SL-FL* did not localize to the PCF (Fig. 4D). These results indicate that the nascent polypeptides translated from *anillin* mRNA are important for its mRNA localization. However, since CDS 1, 2, and 3 are translated into a part of the Anillin protein, translation itself may not have been sufficient for the proper localization of *anillin* mRNA (Supplementary Figs 2C and 3).

**Figure 5:**
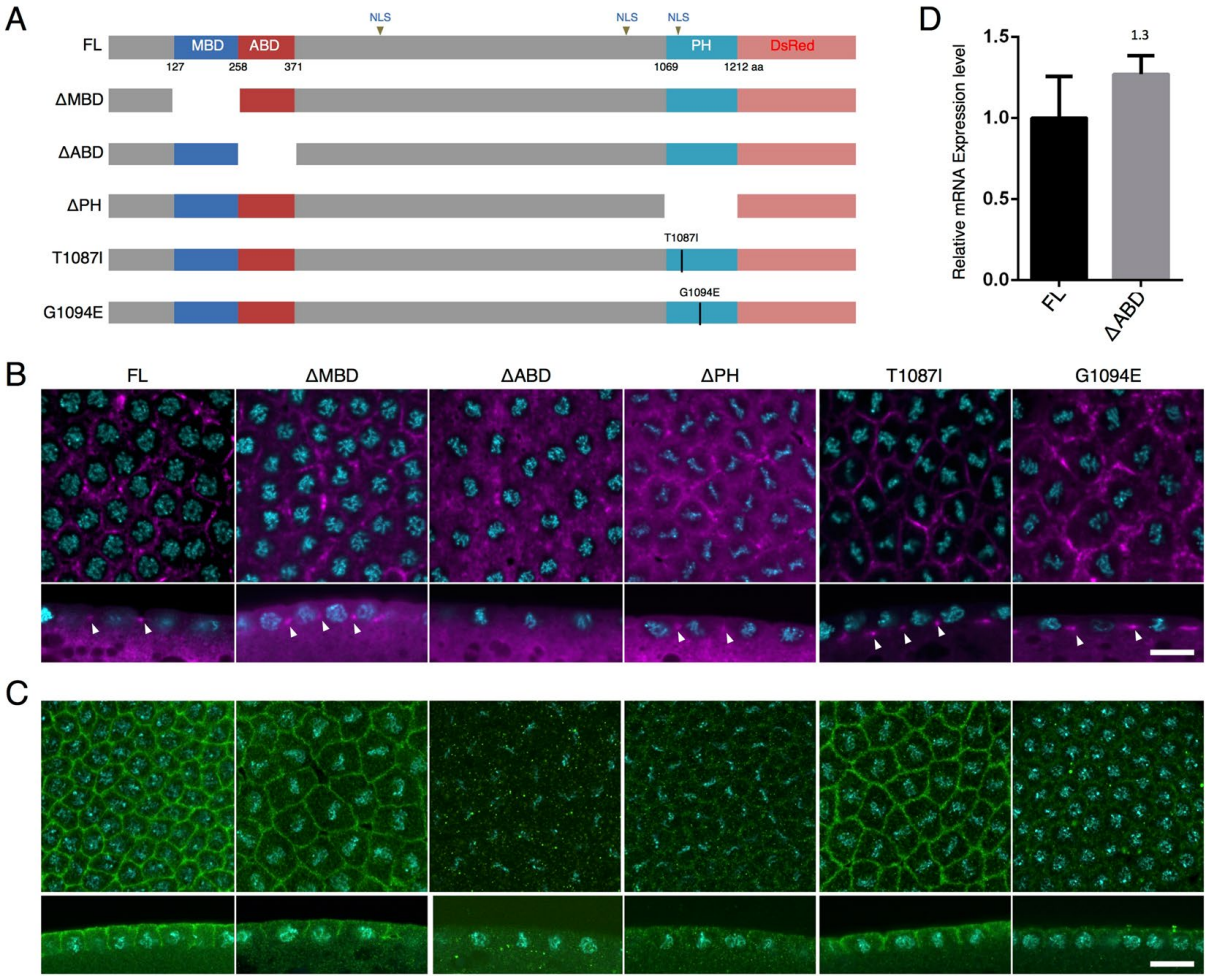
ABD is necessary for the localization of *anillin* mRNA to the tip of the PCF. (**A**) Diagram of deletion or amino acid-substitution variants. (**B**) The localization pattern of each deletion or mutated mRNA was detected with an anti-sense RNA probe against the DsRed-monomer-coding sequence. The localization pattern of each mRNA in at least three different embryos was observed. There was no significant difference among the examined embryos. Each mRNA signal and DNA stained with DAPI is shown in magenta and cyan, respectively. (**C**) The localization pattern of proteins encoded by each deletion or mutated mRNA was detected with anti-RFP IgG. The localization pattern of each fusion protein in at least three different embryos was observed. There was no significant difference among the examined embryos. Each protein signal and DNA stained with DAPI are shown in green and cyan, respectively. Upper and lower panels show horizontal and vertical sections. Each panel is shown at the same magnification. The scale bar indicates 50 µm. (**D**) The expression level of *ΔABD* mRNA was quantified by RT-qPCR and compared to that of FL. Data are means ± SD (n = 3).

Therefore, we examined whether the specific domain of the Anillin protein is required for the localization of *anillin* mRNA to the PCF. Anillin contains three known domains: the MBD, ABD, and PH domain (Fig. 5A)^7,14,23^. In order to investigate the role of each domain region of the Anillin protein in its mRNA localization, we generated transgenic fly lines expressing each domain-deleted Anillin, namely, ΔMBD, ΔABD, and ΔPH (Fig. 5A). In addition to these deletions, we investigated the effects of base substitutions corresponding to each amino acid in the PH domain (T1087I, G1094E) that are suggested to be responsible for PI(4,5)P_2_ binding^16^ (Fig. 5A). PI(4,5)P_2_ has been predicted to function in cell cleavage during cytokinesis^16^. Domain-deleted or base-substituted mRNAs, except for *ΔABD*, localized to the tip of the PCF (Fig. 5B, arrowheads and Supplementary Fig. 2D). Based on RT-qPCR data, the expression level of *ΔABD* appeared to be sufficient for detection even if its mRNA was not specifically localized to the PCF (Fig. 5D). These results demonstrate that ABD is necessary for the proper localization of *anillin* mRNA.

In *ΔMBD* or *T1087I* base-substituted transgene-expressing embryos, those mRNAs and proteins were precisely localized (Fig. 5B,C). However, while *ΔPH* and *G1094E* base-substituted mRNAs localized to the PCF (Fig. 5B), the localization of their proteins to the PCF was very faint (Fig. 5C). The results of a Western blot analysis and its quantified data revealed that the protein level of ΔABD, ΔPH, or G1094E was similar to that of ΔMBD or T1087I (Supplementary Fig. 4). These results suggest that not only ABD, but also the PH domain is required for the proper localization of the Anillin protein in the syncytial blastoderm, as reported for the localization of the human Anillin protein to the cleavage furrow of mammalian cultured cells^23^.

## Discussion

It is well known that subcellular mRNA localization is one of the most important processes to express proper protein function in organisms. However, in contrast to the rapidly increasing number of studies describing subcellularly localized mRNAs, our knowledge on the molecular mechanisms underlying mRNA localization is still limited. In an attempt to reduce this gap, we herein focused on *anillin* mRNA, which shows a characteristic hexagonal localization pattern in the *Drosophila* syncytial blastoderm^12^, and revealed that *anillin* mRNA localizes to the tip of the PCF and interior embryo throughout the cell cycle in the syncytial blastoderm (Lower panels of Fig. 1A’–E”).

The localization patterns of a series of deficient mRNAs indicated that the CDS of *anillin* mRNA is necessary and sufficient for its PCF localization (Fig. 3 and Supplementary Fig. 2B). The *in situ* hybridization analysis of translationally inactive mRNA-expressing embryos revealed that the PCF localization of *anillin* mRNA was achieved in its own translation-dependent manner (Fig. 4). Translation-dependent mRNA localization is widely observed in several different types of cells and organisms. In most cases of co-translational mRNA subcellular localization, the translation of these mRNAs is transiently paused^18,20,24,25^. Regarding *anillin* mRNA, it is important to note that ribosome footprint data with *Drosophila* early embryos (RPFDB: http://sysbio.gzzoc.com/rpfdb/index.html)^26^ match the predicted translational pause or delay occurring downstream of the sequences encoding ABD. Consistent with this finding, 7 successive prolines are downstream of the ABD. Since the polyproline stretch over 6 prolines in the nascent polypeptide was shown to strongly repress its own translation under eIF5A-absent conditions^27^, the translation of *anillin* mRNA may be paused at the polyproline stretch in *anillin* mRNA.

We revealed that nascent polypeptides including ABD of SV-A are necessary for the localization of its mRNA to the tip of the PCF, and *SV-B* mRNA was localized to the furrow tip even when an additional 27 amino acids were inserted into its ABD (CDS in Fig. 3B). A previous study reported that some actin-binding proteins expressed in the syncytial blastoderm were not localized to the PCF^27^. These findings suggest that actin-binding activity may not be sufficient to make its mRNA localize to the tip of the PCF. However, *CDS 1* mRNA does not localize to the furrow tip even though it encodes ABD (Supplementary Fig. 3C). In *S*. *cerevisiae*, *ABP140* mRNA localizes to the distal pole of the mother cell through its own nascent polypeptide, which mainly includes ABD^27^. However, not only ABD, but also additional polypeptides without sequence specificities are needed for the proper localization of its mRNA. As is the case for *ABP140* mRNA, additional polypeptides of the Anillin protein besides ABD may be necessary for the localization of *anillin* mRNA to the tip of the PCF. *ΔPH* mRNA, which is truncated but carries a longer additional sequence than *CDS 1* mRNA, localized to the furrow tip, whereas *CDS 1* mRNA did not (Fig. 5B).

The localization patterns of CDS 1 and 3 revealed that the CDS 1 protein including ABD colocalized with actin at the apical region of the PCF (Supplementary Fig. 3C’–C”’), while the CDS 3 protein may recruit Peanut and narrowly localized to the tip of the PCF, whereas *CDS 3* mRNA did not (Supplementary Fig. 3E–E”’). However, the localization of both of these proteins was not same as that of FL or CDS (Fig. 5C and Supplementary Fig. 3B’,E’). The PCF may form in embryos expressing CDS 1 or CDS 3, and endogenous Anillin protein was also expressed and localized normally (data not shown). Nevertheless, actin and Peanut exhibited abnormal localization patterns from those in wild-type embryos (Supplementary Fig. 3C”,E”)^15,28^. These results suggest that the expression of CDS 1 or CDS 3 exerts a dominant negative effect on the function of endogenous Anillin protein. Furthermore, these results indicate that the precise localization of the Anillin protein in the syncytial blastoderm requires multiple domains, similar to mammalian Anillin (Oegema *et al*.^23^). The localization patterns of ΔABD and ΔPH protein are also consistent with the requirement of multiple domains for the precise Anillin protein localization in the syncytial blastoderm (Fig. 5C). We also revealed that G1094E base-substituted protein shows impaired ability to localize to the tip of the PCF, while T1087I base-substitution does not affect on the Anillin protein localization (Fig. 5C). Although previous study reported that both the glycine at position 1094 and threonine at position 1087 are required for the binding to phosphatidylinositol phosphate lipids including PI(4,5)P_2_ or liposome^16^, the glycine at position 1094 might therefore have another function other than the binding to PIPs that may be important for the Anillin protein localization.

In order to localize the Anillin protein precisely in the syncytial blastoderm, we revealed that it is necessary to localize its mRNA to the tip of the PCF in its nascent polypeptide-dependent manner. However, while *anillin* mRNA in the cellular blastoderm following the syncytial blastoderm does not localize subcellularly to the tip of the invagination, Anillin protein can localize to the furrow tip (Field and Alberts, 1995) (data not shown). These results suggest that other trans-factor(s) is (are) necessary for the localization of anillin protein to the furrow tip in the later cellular blastoderm.

## Materials and Methods

### *Drosophila* stocks

Fly stocks were cultured at 25 °C on standard food containing 0.65% agar, 10% glucose, 5% corn flour, and 3% rice bran. Canton S was used as the wild type. The *nanos*-GAL4 (4937) and the UASp-*GFP*-*scra* (51348) (CDS) flies used in the present study were obtained from the Bloomington Drosophila Stock Center. Transgenic flies carrying UASp-*Full length-DsRed-monomer* (FL), UASp*-∆*5′*UTR-DsRed-monomer* (Δ5′UTR), UASp-*ani-DsRed-monomer-∆CDS* (ΔCDS), UASp*-∆3*′*UTR-DsRed-monomer* (Δ3′UTR), UASp-*stem loop-Full length-DsRed-monomer* (SL-FL), UASp-*CDS*_*1-400aa*_ *-DsRed-monomer* (CDS1), UASp-*CDS*_*401-800aa*_ *-DsRed-monomer* (CDS2), or UASp-*CDS*_*801-1212aa*_ *-DsRed-monomer* (CDS3) were generated using standard methods^15,28^.

### DNA constructs

In order to construct pUASP*-FL-DsRed-monomer*, the *ani* SV-A sequence was amplified with LD23793 (BDGP). FL, Δ5′UTR, ΔCDS, Δ3′UTR, CDS1, CDS2, and CDS3 were cloned into pUASP (Rørth, 1998) in order to generate C-terminal DsRed-monomer fusion proteins. SL-FL was cloned into pUASP, and the stem loop sequence was inserted between 5′UTR and CDS of FL.

### Fixation of *Drosophila* embryos

Embryos were collected for 2.5 hours. They were washed with H_2_O and dechorionated with 10% sodium hypochlorite solution. After washing with H_2_O, embryos were fixed in 1:1 4% paraformaldehyde in phosphate-buffered saline (PBS): heptane with shaking. After washing three times with methanol, embryos were stored at −30 °C and then used for *in situ* hybridization or immunohistochemical staining within one month.

### RNA probe production

PCR was performed to prepare the template for transcription. The PCR products obtained were purified with the High Pure PCR Product Purification Kit (Sigma-Aldrich; 11 732 676 001). Anti-sense RNA probes labeled with digoxigenin (DIG) were synthesized according to the protocol of Lécuyer, E. *et al*.^12^.

### *In situ* hybridization and immunohistochemistry

The conditions and methods for fluorescence *in situ* hybridization and fluorescence *in situ* hybridization combined with immunostaining are described in detail by Lécuyer, E. *et al*.^12,15,28^. The following antibodies were used for fluorescence *in situ* hybridization: Biotin-SP (long spacer) IgG fraction monoclonal mouse anti-Digoxin (1:400; Jackson ImmunoResearch Laboratories Inc.; 200-062-156), Peroxidase IgG fraction monoclonal mouse anti-Biotin (1:100; Jackson ImmunoResearch Laboratories Inc.; 200-032-211), TSA Fluorescein (1:50; PerkinElmer; FP1013), and TSA Reagent Alexa Fluor 594 Tyramide (1:50; Molecular Probes; T20950). The following antibodies were used for immunostaining: rabbit polyclonal anti-anillin IgG (1:500; Provided by Dr. Maria G. Giansanti) (Giansati *et al*., 2015; Sechi *et al*., 2017), mouse anti-dlg IgG (1:100; Developmental Studies Hybridoma Bank (DSHB); 4F3), rabbit polyclonal anti-RFP IgG (1:100; MBL; PM005), mouse monoclonal anti-Lamin IgG (1:100; DSHB; ADL67.10) and mouse monoclonal anti-Peanut IgG (1:100; DSHB; 4C9H4). Actin was stained with Alexa Fluor^TM^ 488 Phalloidin (1:100; Thermo Fisher Scientific; A12379). Samples were inspected with a confocal laser-scanning microscope (Olympus FLUOVIEW FV10i).

### RT-qPCR

RNAs were isolated using TRIzol reagent (Thermo Fisher Scientific). cDNA was synthesized using the PrimeScript RT reagent kit (TaKaRa). PCR was performed with SYBR *Premix Ex Taq* II in the CFX96 Touch^TM^ Real-Time PCR Detection System (BIO-RAD), and data were analyzed with a standard curve-based method calculated with CFX Manager^TM^ software. The specificities of primers were tested with melt curves created by CFX Manager^TM^ software and agarose gel electrophoresis of the amplified fragments. The following primers were used for RT-qPCR: *DsRed-monomer* (fw 5′-ACGGCACCTTCATCTACAAG-3′/rev 5′-CGGCAGTCTTCTTCTGCATTA-3′), *GAPDH* (fw 5′-GGAGCCACCAGACGAAAC-3′/rev 5′-CGAACACAGACGAAGGG-3′).

### Western blotting

Flies laid embryos for 2 hours on embryo collection plates. Embryos dechorionated with 10% sodium hypochlorite were homogenized in a double volume of 2 × Laemmli SDS sample buffer (4% SDS, 20% glycerol, 0.004% BPB 0.125 M Tris-HCl [pH6.8], and 10% 2-mercaptoethanol) and then centrifuged at 14,000 × *g* at 4 °C for 20 min after boiling at 95 °C for 5 min. The supernatants were separated on SDS-polyacrylamide gels containing 6% acrylamide. Western blots were performed using mouse monoclonal anti-α-tubulin IgG (1:8000; Sigma; T5168) or rabbit polyclonal anti-RFP IgG (1:1000; MBL; PM005). Horseradish peroxidase-conjugated secondary antibodies were from Thermo Fisher Scientific (#31430 and #31460). Regarding detection using anti-RFP IgG, primary and secondary antibodies were diluted with Can Get Signal Solution-1 and 2 (TOYOBO), respectively.

## Electronic supplementary material

Supplementary Information
