# Author Correction: The ABD on the nascent polypeptide and PH domain are required for the precise Anillin localization in *Drosophila* syncytial blastoderm

**DOI:** 10.1038/s41598-021-01791-5

**Published:** 2021-11-18

**Authors:** Tomoki Hirashima, Ryo Tanaka, Masamitsu Yamaguchi, Hideki Yoshida

**Affiliations:** 1 Department of Applied Biology, Kyoto Institute of Technology, Matsugasaki, Sakyo-ku, Kyoto, 606-8585 Japan; 2 The Center for Advanced Insect Research, Kyoto Institute of Technology, Matsugasaki, Sakyo-ku, Kyoto, 606-8585 Japan

Correction to: *Scientific Reports* https://doi.org/10.1038/s41598-018-31106-0, published online 27 August 2018

The original version of this Article contained errors where the works of Giansanti (2015) and Sechi (2017) were incorrectly cited in the Materials and Methods section, under subheading ‘In situ hybridization and immunohistochemistry’. As a result, References 29 and 30 were omitted from the Reference list.

“The following antibodies were used for immunostaining: rabbit polyclonal anti-anillin IgG (1:500; Provided by Dr. Maria G. Giansanti) (Giansati *et al*., 2015; Sechi *et al*., 2017), mouse anti-dlg IgG (1:100; Developmental Studies Hybridoma Bank (DSHB); 4F3), rabbit polyclonal anti-RFP IgG (1:100; MBL; PM005), mouse monoclonal anti-Lamin IgG (1:100; DSHB; ADL67.10) and mouse monoclonal anti-Peanut IgG (1:100; DSHB; 4C9H4).”

now reads:

“The following antibodies were used for immunostaining: rabbit polyclonal anti-anillin IgG (1:500; Provided by Dr. Maria G. Giansanti)^29,30^, mouse anti-dlg IgG (1:100; Developmental Studies Hybridoma Bank (DSHB); 4F3), rabbit polyclonal anti-RFP IgG (1:100; MBL; PM005), mouse monoclonal anti-Lamin IgG (1:100; DSHB; ADL67.10) and mouse monoclonal anti-Peanut IgG (1:100; DSHB; 4C9H4).”

The original Article has been corrected.

