# Supplementary material for: The ABD on the nascent polypeptide and PH domain are required for the precise Anillin localization in Drosophila syncytial blastoderm
Source: Sci Rep. 2018 Aug 27;8:12910. doi: 10.1038/s41598-018-31106-0 (PMC6110771; doi:10.1038/s41598-018-31106-0)
Supplement: Supplementary file 1 — Supplementary Information [file 41598_2018_31106_MOESM1_ESM.docx]

**Supplementary information**

**The ABD on the nascent polypeptide and PH domain are required for the precise Anillin localization in *Drosophila* syncytial blastoderm**

Tomoki Hirashima, Ryo Tanaka, Masamitsu Yamaguchi, and Hideki Yoshida

**Supplementary Figure 1.**

**
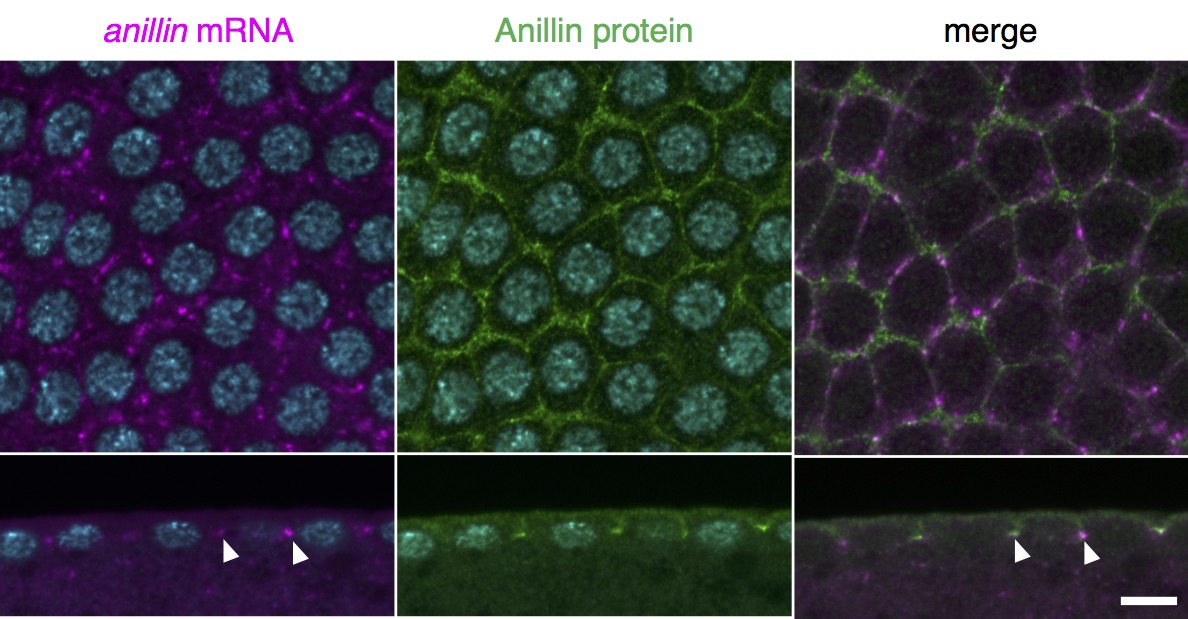
**

**Supplementary Figure 2.**

**
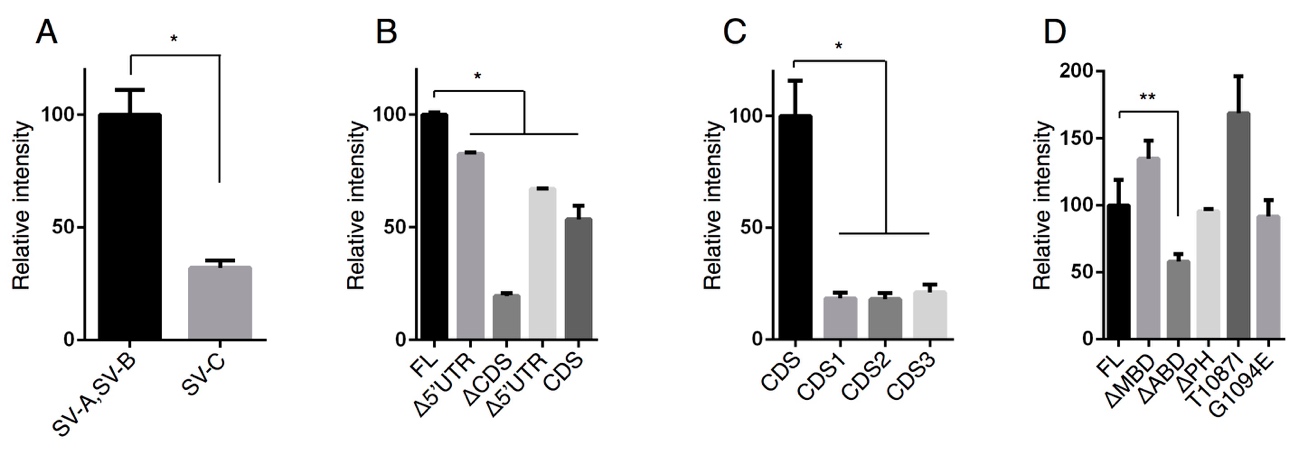
**

**Supplementary Figure 3.**

**
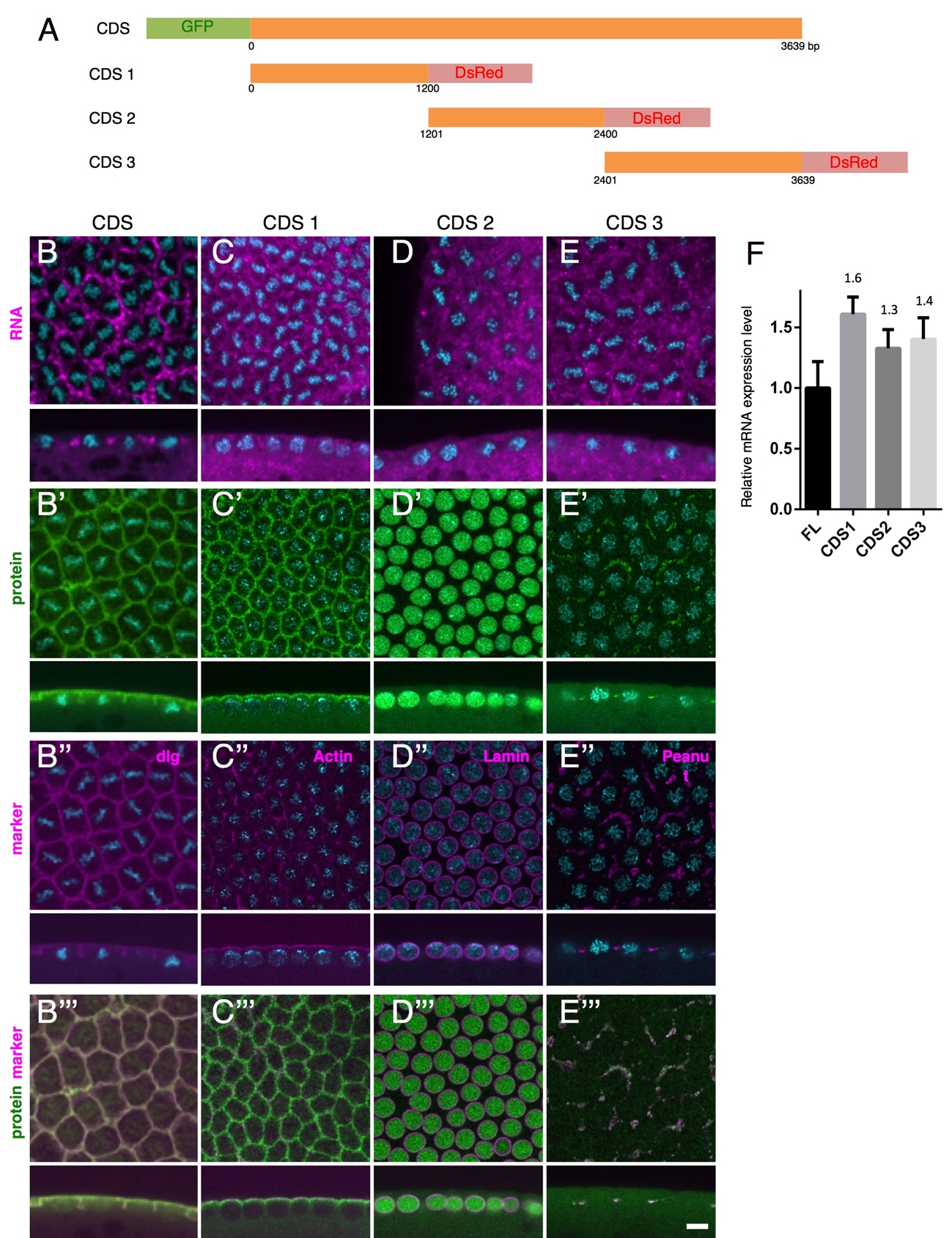
**

**Supplementary Figure 4.**

**
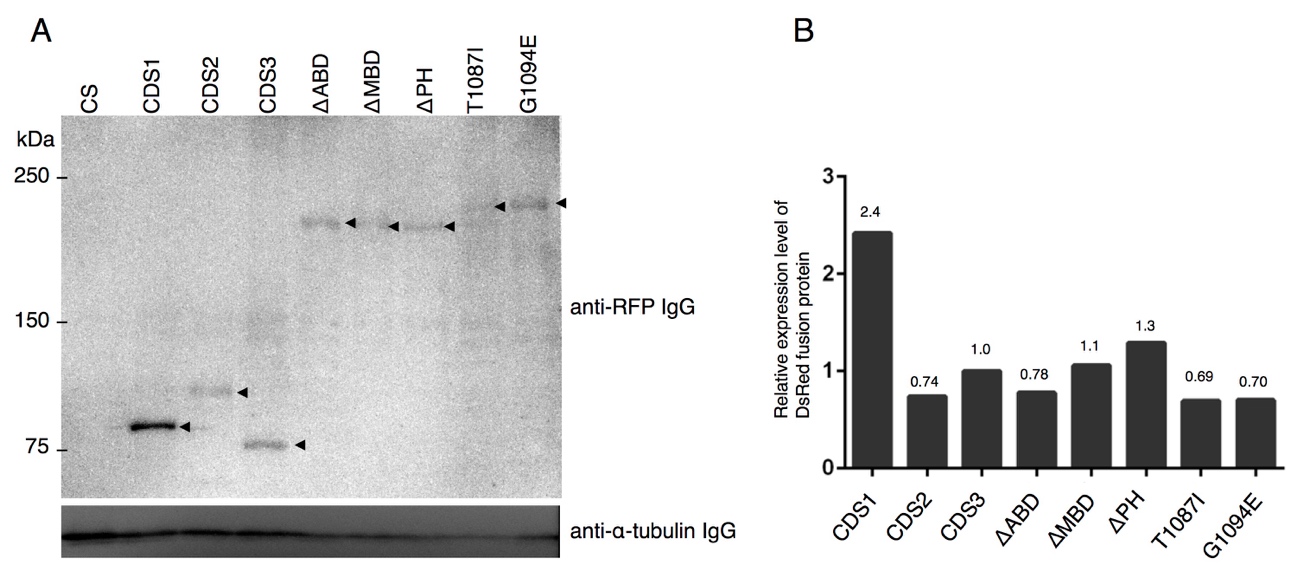
**

**Supplementary Figure 5.**

**
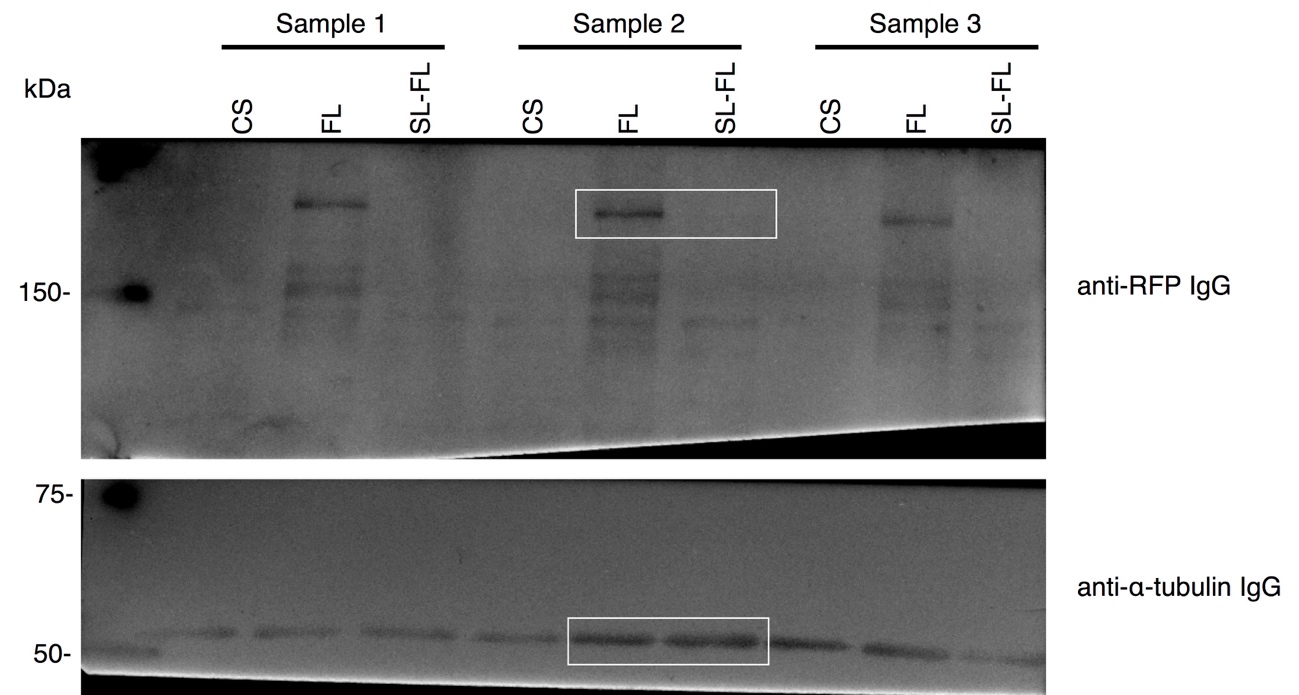
**

**Figure legends**

**Supplementary Figure 1.** Endogenous Anillin protein and mRNA colocalized at the tip of the PCF only. The localization pattern of each *anillin* mRNA or protein was detected with an anti-sense RNA probe against *anillin* mRNA and anti-Anillin IgG, respectively. Each mRNA, DNA, and protein is shown in magenta, cyan, and green, respectively. Merged images of *anillin* mRNA signals (magenta) and anti-Anillin IgG signals (green) are also shown. *anillin* mRNA colocalized with the Anillin protein at the tip of the PCF (arrowheads in the lower panels). The localization patterns of *anillin* mRNA and Anillin protein in at least three different embryos were observed. Upper and lower panels show horizontal and vertical sections, respectively. Each panel is shown at the same magnification. The scale bar indicates 50 µm.

**Supplementary Figure 2.** Quantification of relative intensity of mRNA foci at the tip of the PCF. Quantification of (A) Fig. 2C, (B) Fig. 3B, (C) Supplementary Fig. 2 or (D) Fig. 5B. Only the lower data than those of control mRNAs were quantified. The signal at the tip of the PCF was normalized with the signal of the region below chromosome. When the mRNA does not localize to the tip of the PCF, the signal intensity of the region in the middle between chromosomes at which the tip of the PCF is predicted to form was used for the analysis. The signal intensity at least at three different PCFs was measured. Statistical analysis was performed using t-test. *p < 0.01, **p < 0.05.

**Supplementary Figure 3.** None of the three segments of *SV-A CDS* were sufficient for the localization of *anillin* mRNA to the tip of the PCF. (A) Diagram of the three constructs of CDS. (B-D”) The localization pattern of each segmented mRNA and protein, and of actin, Lamin, and Peanut. (B-D’) Each mRNA, DNA, and protein is shown in magenta, cyan, and green, respectively. (B”-D”’) Actin, Lamin, and Peanut were shown in magenta. Each segmented protein was shown in green. These localization patterns were observed in at least three embryos. There was no significant difference among the examined embryos. Upper and lower panels show horizontal and vertical sections. The scale bar indicates 50 µm. (E) The expression levels of *CDS 1*, *CDS 2*, and *CDS 3* were detected by RT-qPCR and compared to that of FL. Data are means ±SD (n=3).

**Supplementary Figure 4.** All transgenes used in the present study were expressed in the syncytial blastoderm. (A) The protein derived from each transgene was detected by a Western blot analysis with anti-RFP IgG. The arrowheads show that the transgene products were detected as discrete bands of the predicted sizes. (B) Quantification of relative RFP fusion protein expression level. The RFP fusion protein levels were normalized with α-tubulin protein expression levels.

**Supplementary Figure 5.** The full-length blots of Figure 4C. The white boxes represent the area of trimmed panels in Figure 4C.
